# Complete Nucleotide Sequence of an *Escherichia coli* Sequence Type 410 Strain Carrying *bla*_NDM-5_ on an IncF Multidrug Resistance Plasmid and *bla*_OXA-181_ on an IncX3 Plasmid

**DOI:** 10.1128/genomeA.01542-17

**Published:** 2018-02-01

**Authors:** Søren Overballe-Petersen, Louise Roer, Kim Ng, Frank Hansen, Ulrik S. Justesen, Leif P. Andersen, Marc Stegger, Anette M. Hammerum, Henrik Hasman

**Affiliations:** a Department of Bacteria, Parasites and Fungi, Statens Serum Institut, Copenhagen, Denmark; b Department of Clinical Microbiology, Rigshospitalet, Copenhagen, Denmark

## Abstract

Using Nanopore sequencing, we describe here the circular genome of an *Escherichia coli* sequence type 410 (ST410) strain with five closed plasmids. A large 111-kb incompatibility group F (IncF) plasmid harbored *bla*_NDM-5_ and 16 other resistance genes. A 51-kb IncX3 plasmid carried *QnrS1* and *bla*_OXA-181_. *E. coli* isolates with both *bla*_NDM-5_ and *bla*_OXA-181_ carbapenemases are rare.

## GENOME ANNOUNCEMENT

Carbapenems are used for the treatment of serious infections caused by multiresistant *Enterobacteriaceae* strains, such as strains of *E. coli* and *Klebsiella pneumoniae*. The increase in carbapenemase producing *Enterobacteriaceae* worldwide is of great concern. Here, we present the complete genome of the meropenem-resistant *Escherichia coli* strain AMA1167, including its five plasmids, isolated from a liver abscess from a patient hospitalized at Rigshospitalet, Copenhagen, Denmark. The isolate was sent to Statens Serum Institut, Denmark, as part of the national surveillance of carbapenemase-producing organisms (1). Susceptibility testing was performed using the Sensititre GNX3F panel (Thermo Fisher Scientific, East Grinstead, United Kingdom). The isolate was susceptible to amikacin, tigecycline, colistin, polymyxin B, and fosfomycin. Genomic DNA was extracted with the DNeasy blood and tissue kit (Qiagen, Hilden, Germany) and prepared with the Nextera library kit (Illumina, Little Chesterford, United Kingdom) for 251-bp paired-end sequencing (MiSeq, Illumina) according to the manufacturer’s instructions. The data were assembled with CLC bio’s Genomics Workbench version 8.0 (Qiagen, Aarhus, Denmark).

The *E. coli* isolate belonged to sequence type 410 (ST410) based on the multilocus sequence typing (MLST) version 1.8 webserver (2). ResFinder version 2.1 detected two carbapenemase genes, *bla*_NDM-5_ and *bla*_OXA-181_ (3). For further characterization of *E. coli* strain AMA1167, three Nanopore libraries were prepared using (i) a SQK-RAD001 rapid sequencing kit, with 400 ng DNA extracted with a Qiagen plasmid midi kit and run in a FLO-MIN105 R9 flow cell; (ii) a SQK-NSK007 native sequencing kit, with 3 µg DNA; and (iii) a SQK-RAD001 rapid sequencing kit, with 200 ng DNA. For both (i) and (ii), DNA was extracted with a Qiagen genomic-tip 500/G and run in FLO-MIN106 R9.4 flow cells. All three libraries were run in a MinION Mk1B according to the manufacturer’s instructions (Oxford Nanopore Technologies [ONT]) and base called with ONT’s Metrichor software. Fastq files were extracted with Poretools version 0.6.0 (4). Unicycler version 0.4.0 was used for a hybrid assembly of the MiSeq data (5), which was manually corrected using Genomics Workbench version 10.1.1 (Qiagen). The genome was annotated using the Rapid Annotation using Subsystem Technology server (RAST [http://rast.nmpdr.org]).

Plasmid replicons were detected using PlasmidFinder version 1.3, and incompatibility group F (IncF) subtyping was performed using pMLST version 1.4 (6). *bla*_NDM-5_ was detected on a 110,959-bp plasmid (pAMA1167-NDM-5) belonging to IncF and pMLST type [F1:A1:B49]. Besides *bla*_NDM-5_, 16 other resistance genes were detected on pAMA1167-NDM-5, as follows: *bla*_CTX-M-15_, *bla*_OXA-1_, *bla*_TEM-1B_ (two copies), *aadA2*, *aadA5*, *aac(3)-IInd*, *strA*, *strB*, *aac(6′)Ib-cr*, *mph*(A), *catB4*, *sul1* (two copies), *sul2*, *tet*(B), *dfrA12*, and *dfrA17*. The *bla*_OXA-181_ gene was detected on a 51,479-bp IncX3 plasmid (pAMA1167-OXA-181) together with *QnrS1*. The isolate also contained three smaller Col-like plasmids ranging from 1.9 to 2.3 kb (pAMA1167-3, pAMA1167-4, and pAMA1167-5) devoid of any known resistance genes.

Reports of *E. coli* isolates carrying both *bla*_NDM-5_ and *bla*_OXA-181_ are rare, whereas this combination is more common in *K. pneumoniae* isolates (7–9). An *E. coli* ST410 strain with *bla*_NDM-5_ and *bla*_OXA-181_ has been reported from a patient from Egypt (10). The patient in our study had been to Egypt prior to the detection of *E. coli* AMA1167, and thus the strain was likely contracted during his stay in Egypt.

### Accession number(s).

This complete genome project has been deposited at GenBank under the accession numbers CP024801 to CP024806.
